# Telomere biology: from disorders to hematological diseases

**DOI:** 10.3389/fonc.2023.1167848

**Published:** 2023-05-19

**Authors:** Kleoniki Roka, Elena E. Solomou, Antonis Kattamis

**Affiliations:** ^1^ Division of Pediatric Hematology-Oncology, First Department of Pediatrics, National & Kapodistrian University of Athens, “Aghia Sophia” Children’s Hospital, Full Member of ERN GENTURIS, Athens, Greece; ^2^ Department of Internal Medicine, University of Patras Medical School, Rion, Greece

**Keywords:** telomeres, telomere biology disorders, hematological malignancies, pediatric, adult

## Abstract

Variations in the length of telomeres and pathogenic variants involved in telomere length maintenance have been correlated with several human diseases. Recent breakthroughs in telomere biology knowledge have contributed to the identification of illnesses named “telomeropathies” and revealed an association between telomere length and disease outcome. This review emphasizes the biology and physiology aspects of telomeres and describes prototype diseases in which telomeres are implicated in their pathophysiology. We also provide information on the role of telomeres in hematological diseases ranging from bone marrow failure syndromes to acute and chronic leukemias.

## 1 Introduction- historical landmarks of telomeres and telomerase

In the 1930s, H. Muller and B. McClintock reported that while the chromosomal ends were protected from rearrangements and fusion, damaged chromosomes were unstable and susceptible to these events (1–4). Muller used the Hellenic words “telos” and “meros”, interpreted as end and part accordingly; hence ‘end-parts, and proposed the term telomere (3–5). However, the molecular nature of telomeres was unknown at the time.

Hayflick demonstrated in 1961 that human fetal cells possessed a maximum number of potential cellular divisions of 50 to 60 doublings. The cells that reached this limit stopped dividing and became senescent. Hayflick also proved that cells reconstituted from the frozen state remembered the population division level at which they were frozen, and underwent further doubling only up to a predetermined maximum. Hayflick’s findings reversed Carrel’s concept since the early 1900s, regarding the cellular ability to replicate indefinitely. The limited number that a physiological cell population divides before subsequently becoming senescent was named the “Hayflick limit” or replicative senescence (4, 6, 7).

In the early 1970s, Watson and Olovnikow observed asymmetry in linear DNA replication and hypothesized that chromosomal DNA was lost from the ends of the lagging strand with each cellular division while the discharge of the terminal RNA primer led to progressing shortening of chromosomes (4, 8, 9).

Blackburn and Gall were the first to sequence telomeric DNA in the protozoan *Tetrahymena thermophila* in 1978, and a few years later Backburn and Greider discovered the existence of a terminal transferase activity leading to the addition of repeated DNA sequences to the chromosomal ends, named telomerase (10–12).

Since then, more than 35000 peer-reviewed research articles have been published on telomeres, their biology, their relationship with aging, and practically every human disease.

## 2 Telomere biology

### 2.1 Telomeres

**Figure 1:**
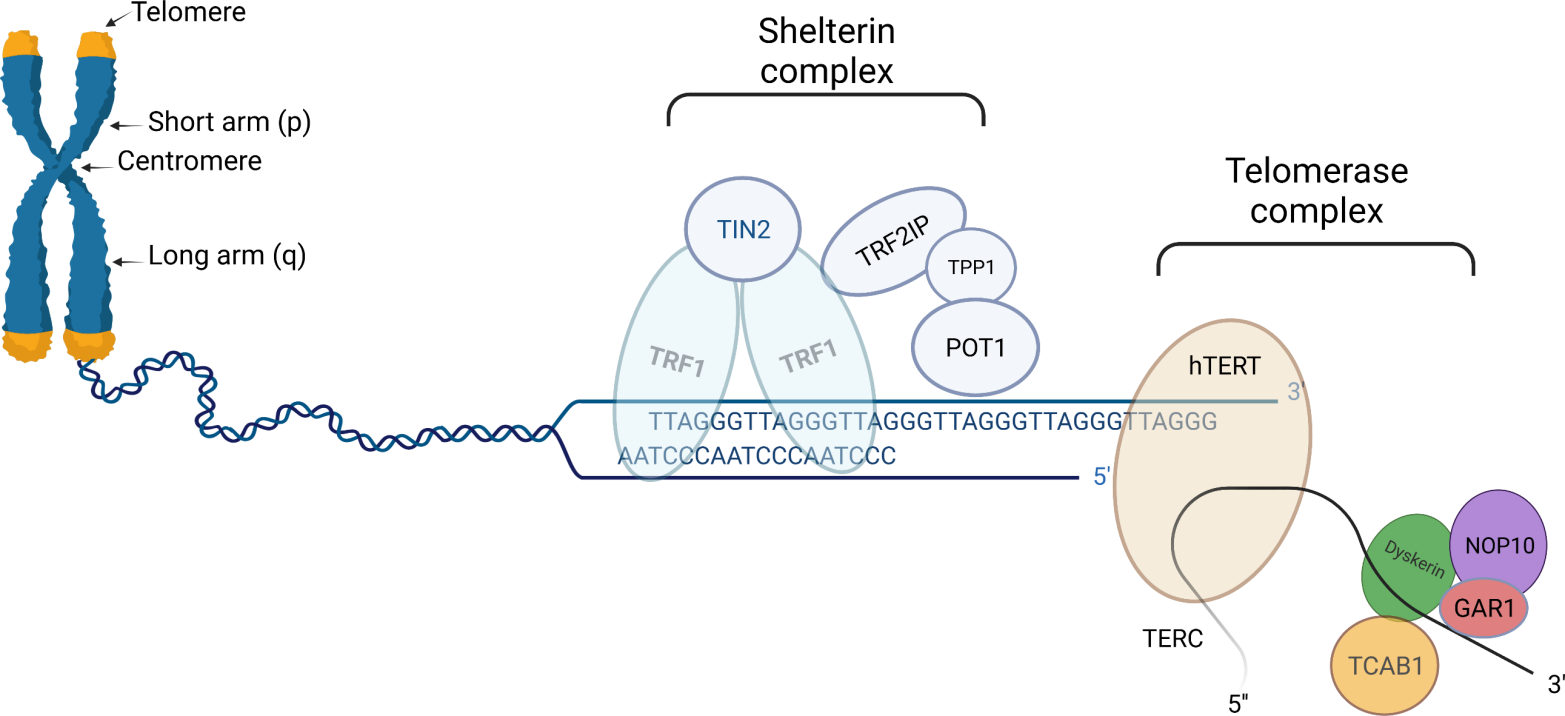
Schematic presentation of telomere (left part), shelterin complex (middle), and telomerase complex (right part). Telomeres are protective structures of tandemly repeated DNA sequence at the ends of eukaryotic chromosomes. In humans, the telomeric DNA sequence consists of repeats of TTAGGG. Shelterin complex is linked to telomeres, helps to stabilize chromosomal ends, and suppresses the DNA damage response system from activating in response to DNA breaks. Human shelterin is specific and consists of six distinct proteinic factors: Telomeric Repeat Binding Factor1(TRF1), TRF2, Repressor/Activator Protein 1 (RAP1), Interacting Nuclear Factor 2 (TIN2), Protector of Telomeres 1 (POT1) as well as POT1-TIN2 organizing protein (TPP1). Telomerase complex preserves critically short telomeres by adding TTAGGG sequences onto the telomeric chromosomal ends; thus contributing by slowing down telomere attrition, although not completely preventing it. Telomerase complex is comprised of a telomerase reverse transcriptase (hTERT) catalytic component, an RNA component (TERC), and several species-specific accessory proteins. In humans, these proteins include dyskerin (DKC1), Nuclear Protein Family A, member 2 (NHP2), Nuclear Protein Family A, member 3 (NOP10), Pontin/Reptin, TCAB1, and Nuclear Protein Family A, member 1(GAR1). “Created with Biorender.com”.

Telomeres are non-coding nucleoprotein structures that protect the ends of eukaryotic chromosomes by discriminating natural ends from regular incidental DNA breaks (**Figure 1**). In this manner, telomeres prevent undesirable end-to-end fusion, nucleolytic degradation, and genomic instability. Without telomeres, every mitosis would result in the loss of genetic information from the leading strand (13).

Telomere length (TL) differs among diverse species (14). Human telomeres are comprised of thousands of tandem repeats of the 5’-TTAGGG-3’ sequence and their length typically ranges between 10 and 15 kilobase pairs (kbp) (13, 14). Although human telomere length has a broad distribution, there are finite upper and lower borders (15).

TL is maintained by a dynamic balance that involves processes of shortening telomeres, such as non-sufficient synthesis of the lagging strand of DNA and end-processing events, as well as processes of telomere lengthening. Only a subset of cell types (germinal, stem, and tumor cells) can avoid progressive telomere shortening through the activation of mechanisms known as “Telomere maintenance mechanisms (TMMs)”. Currently, only two TMMs are known: telomerase-mediated telomere maintenance which is considered the primary TMM, and alternative lengthening of telomeres (ALT) (16–19).

Regardless of the maintenance mechanisms, telomeres shorten by 50-200bp with each cell division (16, 20). Ultimately, repeats loss results in extremely short, dysfunctional telomeres with a loss of capability to adequately cap ends of chromosomes, thus causing impaired cell proliferation, cell senescence, and apoptosis (21).

The time limit at which a chromosome end loses its cap as a result of telomere shortening alters according to a specific rate of each cell type and tissue. Therefore, the shortest telomere is extremely important for cell viability and chromosomal stability (22).

Progressive shortening of TL through loss of telomeric DNA manifests also in the hematopoietic system’s cells as a result of the natural replicative aging process. Since 1994, Vaziri et al. demonstrated that shorter TL was found in human candidate stem cells of adult bone marrow (BM) compared to cells isolated from fetal liver or umbilical cord blood, thus confirming the progressive decline in telomere length with age in hematopoietic stem cells (HSCs) (23, 24).

### 2.2 Telomerase

Telomerase, a reverse transcriptase enzyme, preserves critically short telomeres by elongating chromosome ends *de novo* (**Figure 1**) (25). The increase in TL is achieved through the addition of TTAGGG sequences to the telomeric chromosomal ends. It should be noted, that telomerase although contributing to slowing down this process does not completely prevent telomere attrition (16).

Telomerase is comprised of a telomerase reverse transcriptase (hTERT) catalytic component, an RNA component (TERC), and several species-specific accessory proteins, serving as primers and templates for extending telomeric nucleotide repeats (26–28). The protein known as hTERT functions as a specific reverse transcriptase with conserved catalytic domains. The fundamental non-coding RNA for telomere synthesis, TERC, acts as a template for elongation of the 3′ overhang of the telomeric G-rich strand (18). In humans, the accessory proteins include dyskerin (DKC1), Nuclear Protein Family A, member 2 (NHP2), Nuclear Protein Family A, member 3 (NOP10), Pontin/Reptin, TCAB1, and Nuclear Protein Family A, member 1(GAR1). These proteins have been reported to assist in telomere assembly, trafficking, recruitment, and stability (29).

Telomerase activity (TA) varies throughout human life. The majority of human tissues show a peak in TA expression within the first few weeks of embryogenesis (30). This specific enzyme’s activity is largely repressed starting from the neonatal period, although this is not seen in certain highly proliferative tissues like the skin, intestine, and bone marrow, which are thought to contain stem cell-like subpopulations, or in dividing lymphocytes, ovaries, and testes (31). In mammalian somatic cells and cells with poor proliferative ability, TA is completely absent. Telomerase is also upregulated in the majority of neoplastic cells, which illustrates the importance of telomere maintenance for proliferative potential (32–34).

### 2.3 Telomeric complex

The protein complex known as shelterin, which is linked to telomeres, helps to stabilize chromosomal ends and suppresses the DNA damage response (DDR) system from activating in response to DNA breaks (**Figure 1**). Shelterin’s structure is specific for binding to the structure and sequence of mammalian telomeres. Human shelterin is composed of six distinct protein factors: Telomeric Repeat Binding Factor1 (TRF1), TRF2, Repressor/Activator Protein 1 (RAP1), Interacting Nuclear Factor 2 (TIN2), Protector of Telomeres 1 (POT1) as well as POT1-TIN2 organizing protein (TPP1) (4, 16, 35). Shelterin complex through interacting with TERT binds particularly to telomeric DNA and promotes the telomerase complex to be recruited to chromosomal ends (5, 35).

Another relevant complex is the CST. It consists of three elements: conserved telomere protection component 1 (CTC1), suppressor of cdc thirteen 1 (STN1), and telomeric pathway with STN1 (TEN1). The CST complex partially functions in the synthesis of the telomere-lagging strand (35).

A recently recognized element of the telomere complex is TERRA, a long non-coding telomeric repeat-containing RNA (TERRA) that is transcribed by RNA polymerase II from intrachromosomal telomeric repeats. Although the function of TERRA is not yet fully understood, it is likely to play a role in telomerase regulation, heterochromatin organization in telomeres, gene expression regulation, as well as DDR induced by dysfunctional telomeres (36, 37).

### 2.4 Factors influencing telomere length 

**Figure 2:**
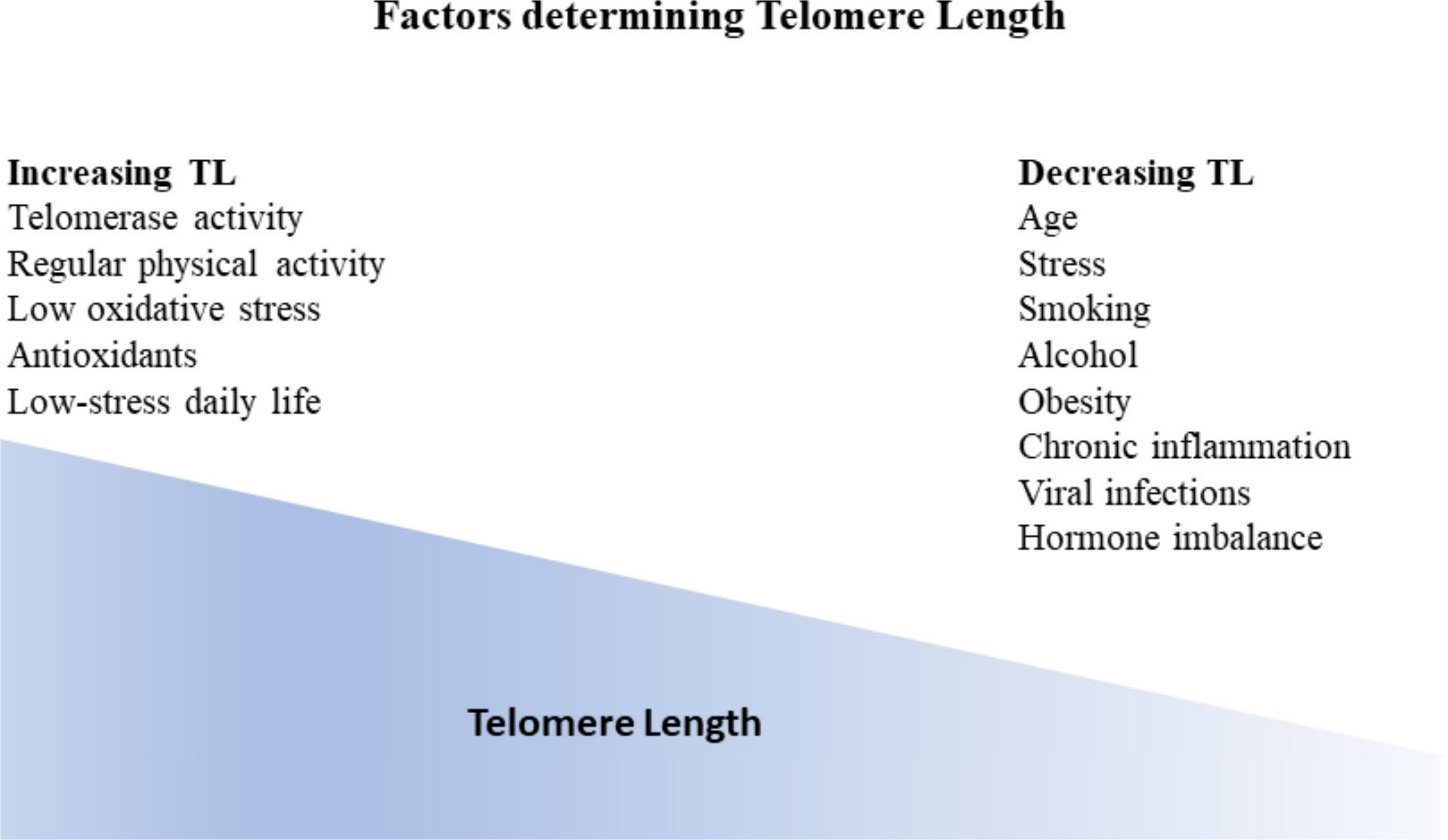
Factors influencing telomere length (TL). Generally, TL is considered a dynamic marker reflecting genetic predisposition as well as individual environmental conditions. TL gradually decreases with age. Additional factors that have been correlated with TL decrease are stress, smoking, alcohol, obesity, chronic inflammation, viral infections as well as hormone imbalance. On the other hand, factors that have been shown to increase telomere length apart from telomerase activity are regular physical activity, low oxidative stress, antioxidants, and low stress daily life.

TL is considered a dynamic marker reflecting genetic predisposition alongside an individual’s environmental conditions (38) (**Figure 2**).

Since the early 1990s, TL has been shown to gradually decrease with age and is considered a sign of human aging (39–41). TL shows a narrow distribution across human populations, ranging from 8 kb to 13 kb in leukocytes from newborns. Telomeres eventually shorten with age normally at a constant rate (5). In contrast to later life, the rate of telomere loss is more pronounced during the first two years of life than during later life (42, 43).

Furthermore, TL is longer in the genomes of African vs. European ancestry (41), females vs. males (44), preterm vs. full-term babies (38), and offspring of older fathers (45–47). Recently, it has been denoted that offspring of older mothers are associated with longer TL (47, 48), thus implying that a joint effect of parental age on TL exists (47, 49, 50). TL also varied among twins and family members, suggesting that extrinsic variables may have an additional impact on TL and attrition (50).

Numerous other factors are correlated with telomere length, named physical activity, smoking, obesity, alcohol use, vitamins, trace elements, chronic inflammation, hormonal replacement therapy, dietary antioxidants, socio-economic status, as well as perceived stress levels (4, 41, 51–55).

Genetic disorders have also been linked to abnormal telomere shortening, involving genes whose dysregulation disrupts the maintenance of telomeres and leads to clinically relevant diseases, named telomeropathies (5).

Recently, Schneider et al. showed that reduced TL was linked to a minor increase in overall mortality risk, although a higher risk of mortality was linked to particular diseases and organs. Malignant tumors, cancer of the esophagus, and lymphoid and myeloid leukemia were considerably more frequent in individuals with shorter TL, despite the lack of an increase in cancer-related deaths (56).

## 3 Telomere biology disorders/telomeropathies

Telomere biology disorders (TBDs) or telomeropathies encompass a broad variety of infrequent disorders that result from inherited defects in telomerase processes or the DDR system (29, 35, 57).

### 3.1 Short telomere syndromes 

**Figure 3:**
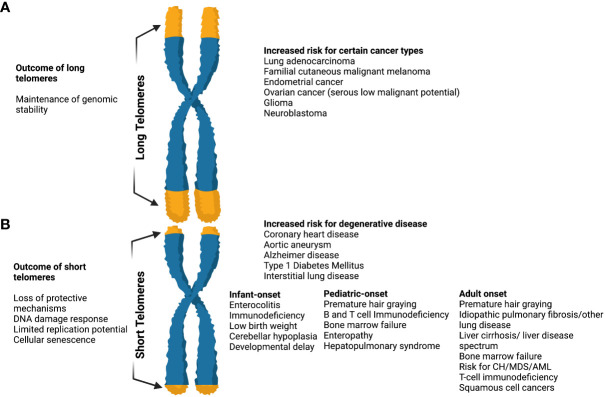
The effects of long and short telomeres. **(A)** Long telomeres are found in some cancer types (i.e. lung adenocarcinoma, familial cutaneous malignant melanoma, endometrial cancer, glioma, etc). They result in the maintenance of genomic instability, although there is increasing evidence that the price of long telomeres is an increased risk of melanoma and glioma. The complete long telomere syndrome spectrum remains incompletely understood. **(B)** Short telomeres result in DNA damage response, loss of protective mechanisms, limited replication potential, and cellular senescence. When present they result in an increased risk of degenerative diseases like coronary heart disease, diabetes mellitus type I, interstitial lung diseases, etc. In short telomere syndromes, different phenotypes are revealed depending on age. The cancer spectrum is notably different when compared to long telomere syndromes and usually involves squamous cell and hematologic malignancies. In general, short telomere syndrome phenotypes can be subclassified in infant, pediatric, and adult onset as shown. “Created with Biorender.com”.

Currently, short telomere syndromes (STS) are considered the best-studied telomere disorder (**Figure 3**). They are accelerated premature aging syndromes that show genetic anticipation and multisystem manifestations (35, 58, 59). Of notice, ancestral generations may present with lung disease whereas their children with bone marrow failure (BMF). Therefore, the entire spectrum of clinical features may appear in a single family.

The severity of telomere shortening determines the type, age of onset, as well as degree of telomere-driven disorders. Thus, STS may present with different manifestations across the age spectrum (58).

The main characteristic that differentiates short telomere syndromes from other Mendelian disorders is the likelihood of unique clinical phenotypes even though all affected individuals share the same genetic abnormality. In general, adults typically develop the disease in slow-turnover tissues, mostly the lung and liver, whereas children and young individuals develop it in high-turnover tissues, i.e. bone marrow (5).

The first disorder to be linked to a pathogenic variant in telomere gene is Dyskeratosis Congenita (DC) (60). It was first described by Zinnser in 1906 in a case report of two brothers with leukoplakia, abnormal skin color, as well as nail dystrophy. Primarily the disorder was thought to be inherited as an X-linked recessive type (XLR). To date, the detection of pathogenic variants in dyskerin (*DKC1*) on the X chromosome leading to reduced TA and shorter TL is the first connection between telomere biology and human illnesses (61).

It has been demonstrated that females are also affected, that the manifestation spectrum is wide and that at least 18 different genes with all inheritance patterns — XLR, autosomal dominant (AD), and autosomal recessive (AR)— are etiologically related (61).

DC is genetically heterogeneous. Currently, directly related pathogenic variants in DC include *DKC1, TERC, TERT, NOP10, NHP2, PARN, RTEL1, TPP1, TCAB1, CTC1, POT1, STN1*, and *TINF2* genes (15, 62).

**Figure 4:**
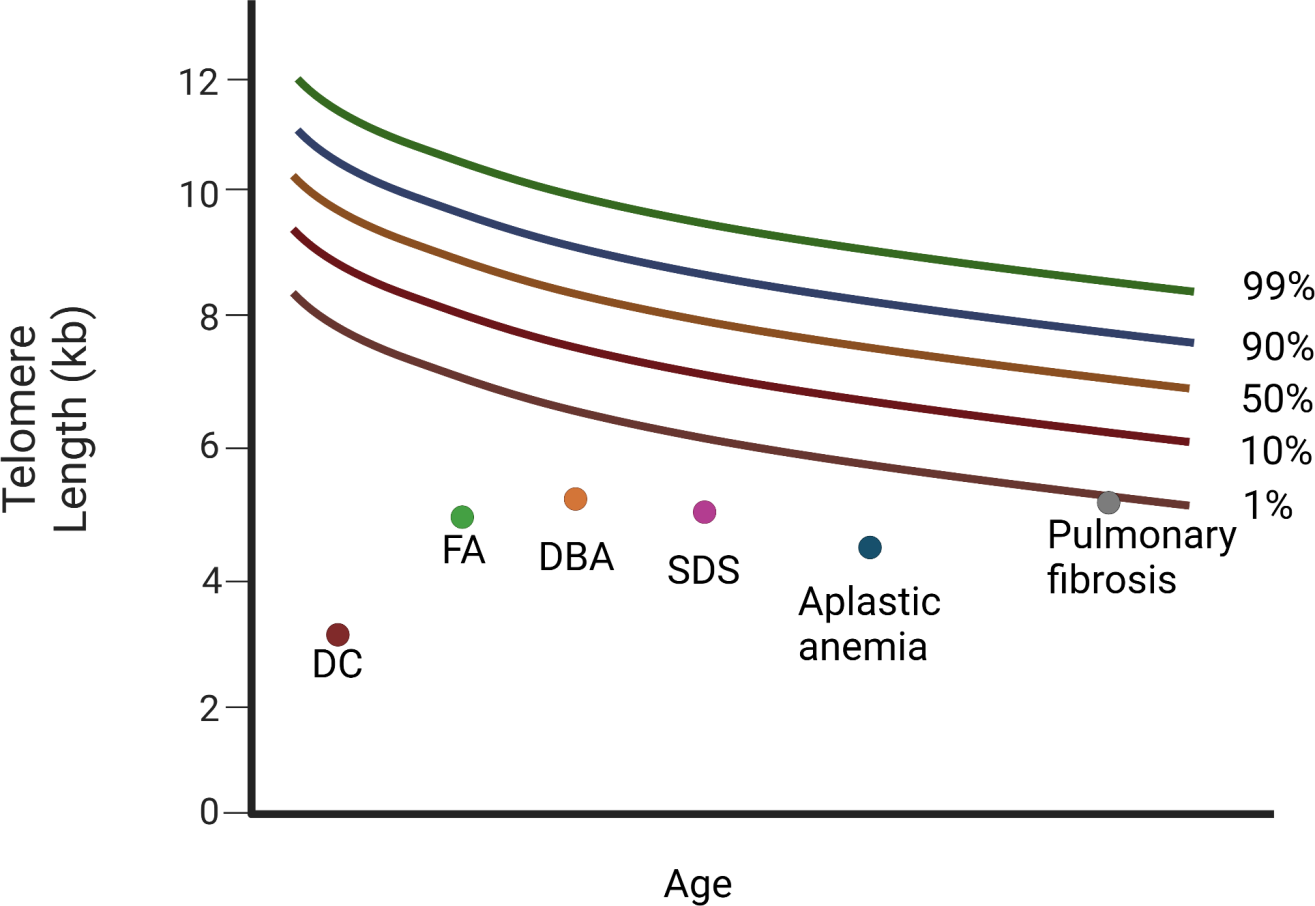
Diagram of relative telomere length (TL) in patients with Dyskeratosis congenita, bone marrow failure syndromes, and the cryptic form of DC, idiopathic pulmonary fibrosis. Different diseases have variable telomere length. Dyskeratosis congenita (DC) is characterised by the shortest telomeres. FA, DBA, and SDS, as well as aplastic anemia (AA), display a mean telomere length below the 1st percentile. Patients with pulmonary fibrosis have telomere length around the 1st percentile (DC, dyskeratosis congenita; FA, Fanconi anemia; DBA, Diamond-Blackfan anemia; SDS, Schwachman-Diamond Syndrome). “Created with Biorender.com”.

Patients with TBDs can be diagnosed using flow FISH lymphocyte telomere lengths that are smaller than the age-specific 1^st^ percentile (**Figure 4**). The ability to distinguish patients with DC from their unaffected relatives using total lymphocyte flow FISH telomere lengths less than the 1^st^ percentile for age had a sensitivity and specificity of 97% and 91%, respectively (61).

#### 3.1.1 Clinical spectrum

The severity of telomere shortening directly affects the severity of symptoms in DC. DC manifests between 15 and 25 years of age in the less severe cases of telomere shortening (TL less than 10% percentile), whereas in the most severe cases (i.e., those with greater telomere shortening and TL less than 1% compared to healthy controls), the age of onset of symptoms is within the first 10 years of life or even during pregnancy. Depending on the mutated gene, DC may appear in a less typical way, that is, bone marrow aplasia or pulmonary fibrosis only (63).

Mucocutaneous triad-nail dystrophy, mucosal leukoplakia, and abnormal skin pigmentation- characterize the classic form of DC (64). Currently, minimal clinical criteria have been established for DC diagnosis. They consist of at least two of the four major characteristics, (abnormal skin pigmentation, nail dystrophy, leukoplakia, and BMF), and at least two of the other extra-hematopoietic manifestations known to occur in DC (**Table 1**) (64).

**Table 1:** Major and minimal clinical criteria for diagnosis of Dyskeratosis Congenita.

Clinical feature/abnormality	% of pts
Major/common features	
Abnormal skin pigmentation	89
Nail dystrophy	88
Bone marrow failure	85.5
Leucoplakia	78
Other recognized somatic features	
Epiphora	30.5
Learning difficulties/developmental delay/mental retardation	25.4
Pulmonary disease/fibrosis	20.3
Short stature	19.5
Extensive dental caries/loss	16.9
Esophageal stricture	16.9
Premature hair loss/greying/sparse eyelashes	16.1
Hyperhiderosis	15.3
Malignancy (Solid tumors/Acute myeloid leukemia)	9.8
Intrauterine growth retardation	7.6
Liver disease/peptic ulceration/enteropathy	7.3
Ataxia/cerebellar hypoplasia	6.8
Hypogonadism/undescended testes	5.9
Microcephaly	5.9
Urethral stricture/phimosis	5.1
Osteoporosis/aseptic necrosis/scoliosis	5.1
Deafness	0.8

[Adopted from Dokal et al. (64)] (62, 65).

Apart from the classical forms of DC, severe forms have been described. Patients with Hoyeraal-Hreidarsson syndrome (HH; OMIM#305000)) present with intrauterine growth retardation, microcephaly, cerebellar hypoplasia, developmental delay, immunodeficiency, and BMF. Patients with Revesz syndrome (RS; OMIM#268130) additionally present with bilateral exudative retinopathy. Finally, patients with Coats plus syndrome (CRMCC; OMIM#612199) may additionally present with intracranial calcifications, brain cysts, leukodystrophy, cerebroretinal microangiopathy, osteopenia, bone fractures, and poor bone healing. These patients may also present gastrointestinal bleeding (62, 66).

Furthermore, it has been demonstrated that some individuals experience a cryptic form of DC marked by delayed symptoms in adulthood (67). This cryptic telomeropathy (cryptic DC) is a late-onset variant that manifests in early adulthood with diverging oligosymptomatic characteristics (62). Isolated aplastic anemia (AA), myelodysplastic syndrome (MDS), acute myelogenous leukemia (AML), cirrhosis of the liver, interstitial lung disease, or a combination of these are common manifestations in patients with cryptic DC (68, 69). The predominant adult-onset presentation of STS, idiopathic pulmonary fibrosis (IPF), is an age-related lung scarring disorder, often identified after the sixth decade (70). In the last few years, it is obvious that the number of individuals that might be considered to have DC has grown considerably. Thus, terms such as “ telomeropathies,” “short telomere syndromes,” and “telomere biology disorders “have been introduced to describe these patients (29). Up to date, individuals with features that fall into the following categories can be considered DC patients:

1. Patients carrying the three defining mucocutaneous characteristics,
2. Patients who exhibit one out of three mucocutaneous characteristics, BMF, or other somatic characteristics of DC,
3. Patients presenting AA or MDS or pulmonary fibrosis linked with a pathogenic telomerase variant,
4. Patients who exhibit four or more features of the Hoyeraal– Hreidarsson syndrome (growth retardation, developmental delay, microcephaly, BMF, immunodeficiency, and cerebellar hypoplasia), or
5. Patients who exhibit two or more DC symptoms, linked to very short telomeres (less than 1^st^ percentile compared to healthy controls) (64).

#### 3.1.2 Propensity to develop hematological disorders and cancer

A common manifestation of TBD is BMF. Individuals with classical DC are almost 80% likely to have at least one single lineage of cytopenia by the age of 30. One cell lineage may initially be altered, but it can subsequently turn into severe pancytopenia or MDS (68). A prospective study showed that by the age of 50 years, 50% of individuals had clinically relevant BMF, and 20% had evident MDS. In some individuals, aplastic anemia may develop, even in the absence of DC-associated symptoms (65, 68).

Apart from genome instability, short telomeres have been shown to promote clonal hematopoiesis (CH), thus providing another paradigm of leukemogenesis (5). It is well-established that human DNA changes with age. Somatic mutations in the same genes associated with MDS and AML are also associated with advanced age. These mutations define CH. Clonal hematopoiesis of indeterminate potential (CHIP) is characterized by the presence of somatic variants in the peripheral blood with a variant allele frequency (VAF) ≥2% in genes associated with hematological malignancies (71). These variants occur more frequently in advanced age (~ 10% at age> 65 years). That said, recent data show that young patients with CHIP are at an increased risk of developing not only hematological malignancies but also coronary heart disease, cardiovascular events, and stroke (72, 73).

Short telomeres are also connected with CHIP; adults with short telomere syndromes have an increased possibility of mutations in the same genes implicated in CHIP. Thus far, data suggest that telomere shortening associated with pathogenic variants in telomerase and telomere-related genes can give rise to CHIP in these patients (74, 75). Schratz et al, reported that 12.8% of patients with short telomere syndromes had cancer, of whom 75% were diagnosed with MDS/AML whereas 30% of adult patients without MDS/AML had age-related CH (74). This percentage was significantly higher than that in patients without TBD, even at older ages (6-10%) (74). Furthermore, in a larger study of 120 patients with TBD, CH-related mutations were found in 48% and specific pathogenic variants correlated with specific somatic mutations. Indeed, it was shown that somatic *POT1* mutations were strongly associated with the *TINF2* pathogenic variant, whereas both *TERTp* and *PPM1D*, were mostly detected in *TERT/TERC* patients with multi-organ disease (76, 77).

The spectrum of solid malignancies associated with STS overlaps with tumors that develop in individuals with T-cell immunodeficiency, including those with acquired immunodeficiency syndrome (AIDS) and those who have received solid organ transplants. To date, every aspect of T-cell development is limited by short telomeres, both in individuals with short telomere syndromes and in mouse models (5).

Alter et al. reported a 4.2 O/E ratio for any malignancy in non-transplanted DC patients. Notably, the O/E ratio for the development of head and neck squamous cell carcinoma (HNSCC), specifically in the tongue, was extremely high (74 and 216, respectively). Concerning hematological disorders, these patients had O/E ratios of 578 and 73 to develop MDS and AML, respectively. Moreover, in DC patients following transplantation the O/E ratio for the development of any malignancy, HNSCC in general, and HNSCC in the tongue was even higher (30, 432, and 1561, respectively). Transplanted DC patients also had an increased possibility of developing Hodgkin (HD) or non-Hodgkin lymphoma (NHL) (78).

#### 3.1.3 Special considerations

Considering the significant risk of cancer, particularly as individuals reach adulthood, it is advised to avoid smoking, exposure to the sun, and alcohol use. Additionally, routine screenings for both hematological and non-hematological malignancies should be performed. Although cancer treatment depends on the specific type, it should take into account the underlying genetic defects (i.e., the need for increased supportive care and reduction in drug dosages) (79).

Early mortality of DC is mainly caused by BMF. It should be noted that patients with DC and BMF showed no response to immunosuppressives (80, 81). According to recent research, sex hormones may increase TA, by acting on the *TERT* gene. Thus, the anabolic steroids danazol and oxymetholone can improve hematopoietic function. The wide phenotypic spectrum of DC, especially silent carriers, may complicate the selection of related stem cell donors (82). Currently, the only long-term treatment for hematopoietic manifestations linked to DC is allogeneic hematopoietic stem cell transplantation (HSCT) although there is an increased risk of pulmonary/vascular and liver complications (83). Finally, non-myeloablative fludarabine-based protocols have decreased the frequent and severe adverse effects observed with standard myeloablative conditioning regimens (65, 79, 83).

### 3.2 Long telomere syndromes 

Studies of primary cultured fibroblasts, in which cells with longer telomeres showed longer replicative potential, supported the evidence that longer TL provides a longevity advantage (54). Furthermore, exogenous TERT expression was sufficient to bypass cellular senescence and immortalize primary cells (55). Recent evidence has supported a greater role for long telomeres in promoting age-related cancer risk, in contrast to observations supporting a low penetrance and narrow spectrum of cancer associated with short telomeres (5) (**Figure 3**).

In humans, upregulation of telomerase was initially related to an increased risk of familial cancer in a five-generation autosomal dominant family with cutaneous malignant melanoma (CMM) which was found to carry a pathogenic variant in the *TERT* promoter (15).

Additionally, pathogenic variants in five telomere-related genes (*TERT* promoter and shelterin genes *POT1, TPP1, TERF2IP*, and *TINF2*) showing autosomal dominant inheritance have been linked to the risk of familial melanoma, glioma, and chronic lymphocytic leukemia, explaining 1-10% of families with such cancer types (5, 84, 85). Haycock et al, in the Mendelian Randomization study, reported that increased TL due to germline genetic variation was widely correlated with elevated risk for site-specific cancers. Notably, the observed associations were stronger for rare cancers and tissue sites characterized by lower rates of stem cell division, such as glioma, serous low-malignant potential ovarian cancer, lung adenocarcinoma, neuroblastoma, bladder cancer, and melanoma (84). In the same study, the risk of non-neoplastic or degenerative diseases was reduced (84).

## 4 Hematological diseases and telomeres

### 4.1 Inherited bone marrow failure syndromes

IBMFs comprise a set of diverse diseases, in which the production of one or more blood cell lines fails (86, 87). Clinical manifestations vary depending on the type and number of blood cell lines involved and may include various combinations of anemia, leukopenia, and/or thrombocytopenia (86–88). Despite having heterogeneous phenotypes and various underlying pathogenetic mechanisms, all IBMFs have a higher risk of MDS and/or AML development. Among all patients with IBMF, those with DC and Fanconi anemia (FA, OMIM#227650) presented the highest risk (**Figure 4**) (86).

Several investigators have demonstrated that apart from DC patients, TL is extremely short in subjects with AA (81) and non-DC IBMFS, such as FA, Schwachman-Diamond syndrome (SDS, OMIM#260400), as well as Diamond-Blackfan anemia (DBA, OMIM#105650) (89, 90).

FA, the most prevalent form of IBMFs, is a rare, multisystemic, hereditary disease characterized by a high frequency of spontaneous chromosomal breakage. This results in genomic instability, which impairs DNA repair and cell-cycle regulation (82, 91, 92). Individuals with FA show hypersensitivity to DNA cross-linking agents (92, 93).

FA is caused by germline pathogenic variants in genes involved in the FA/Breast Cancer Susceptibility (BRCA) DNA repair pathway. Currently, pathogenic variants in 22 genes whose protein products collaborate in the FA/BRCA pathway have been detected in individuals with FA, the most commonly found of which are *FANCA*, *FANCC*, and *FANCG* (94–96).

Shorter telomeres, telomere loss/breaks, and high levels of telomerase activity are typically found in lymphocytes of FA patients (97). To date, it has been suggested that telomere shortening in patients with FA correlates with the progression of bone marrow aplasia (98, 99). In fact, Li et al. demonstrated that the vast majority of patients with FA and severe BMF had higher telomere shortening rates compared to those with FA and on severe BMF (99).

Increased chromosomal breakage, excessive free radical production, and/or abnormal regulation of cell proliferation-related to the deregulation of apoptosis are some of the hypothesized causes of rapid telomere shortening observed in FA (82, 97, 100). Shah et al. recently reported different TL shortening among different complementation groups, with the FANCL group showing the most severe (97). Finally, it has been proposed that the very short telomeres observed in individuals with FA might be the result of hematopoietic failure, stress, and/or treatment (82).

SDS is an autosomal recessive disease characterized by neutropenia, insufficient pancreatic exocrine function, and extra-hematopoietic manifestations, particularly metaphyseal dysostosis (79, 101). The vast majority of individuals with SDS, although not all have been found to harbor pathogenic variants only in the *SBDS* gene (102, 103).

Thornley et al, reported that SDS patients had a considerably reduced mean telomere length adjusted to age compared to healthy controls (p<0.0001) and concluded that stem cell hyperproliferation is implicated for the shorter TL (104). It should be noted that TL in patients with SDS is not as short as that in DC patients (90, 104). In 2018 Liu et al, showed that the underlying mechanism of telomere shortening found in SDS patients is a cell cycle-dependent telomere-protecting protein named SBDS which enables TPP1-mediated telomerase recruitment (102).

DBA is the most common cause of inherited isolated failure in red blood cell production. Most patients show transfusion-dependent anemia in the first year of life, and approximately half of the patients have congenital deformities such as dysmorphic features, short stature, and abnormalities of the eyes, kidneys, and hands (101, 105).

DBA has an autosomal dominant mode of inheritance. Pathogenic variants in genes encoding essential parts of the small 40S or large 60S ribosomal subunits (*RPS19*, most often, *RPS24, RPS1, RPS15, RPS27A, RPS10, RPS29, RPS26, RPL5, RPL11, RPL35A*, as well as *RPL15*) result to a defect in the ribosomal RNA maturation (101). Currently, pathogenic variants in 20 ribosomal protein (RP) genes have been linked to DBA (105).

TL in patients with DBA is not as short as that in patients with DC and seems to be closer to that in healthy controls (90, 106).

### 4.2 Aplastic anemia

AA was initially described by Ehrlich in 1888, and almost all sporadic cases implicate immune pathophysiology. The improvement in blood counts following immunosuppressive treatment (IST) observed in AA patients is the strongest and most pertinent evidence for the involvement of an immune mechanism (107).

According to different studies, the telomere length of leukocytes from patients with AA shows a wide variation, with a greater percentage of patients having shorter telomeres than healthy controls (108, 109).

Short telomeres in patients with acquired severe aplastic anemia were initially thought to result from hematopoietic stress (110). Currently, it is believed that the destruction of HSPCs by the patient’s immune system results in increased mitotic demand on a limited pool of stem cells, and subsequently a probable reduction in TL (107, 111).

Although telomere shortening in marrow failure is caused by loss-of-function mutations in the telomerase complex (*TERC* and *TERT*) genes, it was later found that these mutations were also observed in patients without DC. A minority of patients with acquired aplastic anemia harbor telomerase mutations and show short telomeres (112, 113).

It is noteworthy that Calado et al. found that although the TL of individuals at diagnosis of AA was comparable to that of age-matched subjects, it was conversely associated with the likelihood of developing a cytogenetically aberrant clone. Furthermore, it was shown that the percentage of monosomy-7 cells in the BM at diagnosis of AA was negatively associated with telomere length years before the development of an autonomous and clinically detectable abnormal clone (111).

Ball et al. first reported and confirmed by others that patients with AA had considerably shorter telomeres than controls and that in patients who had a full hematological recovery, the rate of telomere shortening returned to normal for age-related loss (108, 114).

Multivariate analysis in a single institution of 183 patients with severe aplastic anemia (SAA) demonstrated that TL was associated with relapse, clonal evolution, and mortality (110). A major unfavorable event in SAA is the clonal evolution to AML (115). Very short telomeres are the main risk factors for malignant clonal evolution. In fact, patients with shorter telomeres in the SAA cohort evolved to monosomy 7 at a rate that was approximately five to seven-fold higher rate than those with longer telomeres (95% CI, 8.7–37.5%) (110). According to these findings, patients undergoing clonal evolution have accelerated telomere attrition; thus, telomere length measurement can be used to identify patients with SAA at risk (110). Moreover, such findings suggest that in the near future risk stratification and eventually, management of medical treatment may depend on the ability to prognosticate severe disease-related complications (110).

Notably, AA subjects responding to immunosuppressive treatment had TL similar to that of healthy controls, whereas non-responders had considerably shorter telomeres (23). The TL and the number of reticulocytes at diagnosis can predict response to IST (110).

#### 4.2.1 Aplastic anemia in children and telomeres

Children with AA also have significantly shorter telomeres compared to healthy controls. Telomere length appeared to be a significant predictive variable in multivariate analysis for response to IST (81, 116).

### 4.3 Myelodysplastic syndromes

MDS consist of numerous clonal disorders deriving from multipotent hematopoietic stem cells. Ineffective hematopoiesis associated with peripheral blood cytopenia and bone marrow morphologic abnormalities are the main characteristics of MDS. Approximately 1/3 of patients with MDS may progress to acute leukemia (117). Nowadays, the only potentially curative therapy for MDS patients is allogeneic HSCT, although long-term survival is limited by transplant-related risk for complications (118, 119).

Telomere length showed heterogeneity in MDS. Reduced TL was observed in approximately 50% of patients with MDS versus age-matched controls (16, 120, 121). Additionally, reduction in TL seems to be more marked in MDS in transformation (120, 122), in patients with complex abnormalities in the karyotype (120), in patients with more severe pretransplant cytopenias (118), and also in patients with a greater risk international prognostic scoring system (IPSS) or MDS WHO classification-based prognostic scoring system (WPSS) vs those with lower risk (121, 123). Telomere shortening may also be indicative of a higher risk for leukemic transformation (124), and poor prognosis (125, 126).

Regarding telomerase activity, a study showed that upregulation of telomerase is unusual in MDS patients, despite telomere shortening that occurs in almost 40% of patients with MDS at diagnosis (122). According to Park et al. increased TA may be a poor prognostic indicator in MDS, as individuals with a high TA had a statistically significant shorter interval before evolving to acute leukemia (p = 0.010) (127).

Recently, Myllymäki et al. reported that recipient blood TL proved to be a significant predictor of survival and non-relapse mortality (NRM) after HSCT in MDS patients aged>40 years, regardless of established clinical parameters like age, somatic mutations, hematologic factors, treatment history for MDS, performance status, comorbidity index, as well as donor-recipient HLA matching. In the same study, mutations in three genes, *SRSF2* (P=0.001), DNA methyltransferase 3A (*DNMT3A*; P=0.001), and *STAG2* (P=0.04) were more frequently found in individuals with the longest quartile blood TL versus those with the shortest quartile TL. In contrast, three different gene mutations were considerably more frequent in individuals with the shortest telomere length: *PPM1D* (P=0.01), *WT1* (P=0.03), and *ATM* (P=0.04). Furthermore, it was shown that individuals with severe acute graft-versus-host disease (GVHD) had the greatest effects of TL on NRM and were more prominent after high-intensity conditioning (118).

Interestingly, 2.7% of MDS patients who underwent allogeneic transplantation and did not have a clinical diagnosis of TBD had uncommon *TERT* variants according to a large study by Reilly et al. These polymorphisms were related to shorter TL, younger age at MDS diagnosis, and a higher risk of NRM in MDS patients following allogeneic SCT (128). Multivariate analysis revealed that these uncommon *TERT* uncommon variants were linked to poorer overall survival (OS) (P=0.034), which was caused by a higher incidence of NRM (P= 0.015). These results imply that uncommon *TERT* variants may help in the identification of a subset of MDS patients with undiagnosed telomere biology disorders (128).

#### 4.3.1 Myelodysplastic syndromes in children and telomeres

Children with MDS tend to exhibit short telomeres (129). Children show considerable differences in terms of morphology, cytogenetics, and therapeutic approaches compared with adults. The mutational landscape is also different between children and adults with MDS. The implementation of specific molecular testing and NGS in daily practice is expected to identify more children with MDS and an underlying genetic predisposition syndrome (130).

### 4.4 Hematologic malignancies

Since the early 2000s, several researchers have studied differences in both TL and TA in patients with leukemia (131, 132).

#### 4.4.1 Acute leukemias

Leukemic cells have shorter TL compared to bone marrow mononuclear cells and in subjects with B-cell acute lymphoblastic leukemia (B-ALL) vs T-cell acute lymphoblastic leukemia (T-ALL) vs acute myelogenous leukemia (AML). Telomerase activity was found to be higher in B-ALL vs T-ALL vs AML patients (133). Moreover cytogenetic abnormalities were associated with shorter telomeres in both ALL and AML (133, 134).

##### 4.4.1.1 Acute myelogenous leukemia

Shorter TL is found in adults with AML vs matched controls (median: -2.5TFU; p<0.001) (134), with aberrant karyotype vs normal karyotype (median: -3.0vs -2.3TFU; p<0.03) (134), with multiple aberrations vs less than two aberrations (median: -3.7 vs -2.9 TFU; p<0.03), and younger than 60 years vs older than 60 years (median: -4.3 vs-1.7 TFU; p<0.001) (134, 135).

Noteworthy, individuals diagnosed with AML monocytic subtype M5 according to the FAB classification system, showed the shortest telomeres (-3.8 TFU); versus individuals with M1 (-1.9 TFU, p<0.001) (134). Gaffari et al. reported that patients with acute promyelocytic leukemia (M3) have a significantly shorter TL compared to the control group (median 3.5 versus 11.37 kbp; p < 0.001). The same study demonstrated that all patients had higher TA and that TA was noticeably higher in relapsed group vs the group that had just been diagnosed (136).

It has also been reported that activating mutations like *FLT3-ITD* have been linked to shorter TL in patients (135–137), whereas mutations in epigenetic modifying enzymes, mainly *IDH1* and *IDH2*, have been linked to longer TL in patients (137). Dratwa et al. observed that patients in various risk stratification categories for *FLT3-ITD* and *NPM1* showed different TL and OS rates. The shortest telomeres and noticeably worse OS were seen in AML individuals with *FLT3-ITD* mutations and without *NPM1* mutation (135). Moreover, shorter pre-treatment TL was associated with an inferior relapse-free, event-free, and OS in intermediate-risk AML (138).

Of interest, in a recent study of 5207 patients with AML from the German Study Alliance Leukemia registry, patients younger than 36 years with aberrant karyotype were screened for underlying germline predisposition syndromes named “Myeloid neoplasms with germ line predisposition (MNGLP)” which was detected in 34.5% of them. Patients harboring pathogenic variants in MNGLP-associated genes displayed a markedly decreased OS. Thus, researchers proposed genetic screening for underlying hereditary cancer predisposition syndromes in young patients with AML and aberrant karyotype, as well as the implementation of different treatment protocols to reduce toxicity and improve response to therapy (139).

###### 4.4.1.1.1 Pediatric acute myelogenous leukemia and telomeres

Children with AML have very short TL compared to healthy controls (134, 140). Variants in the telomerase complex genes *TERT* and *TERC* do not appear to increase the risk of developing pediatric AML, and these mutations are rather uncommon in pediatric AML (140).

It should be noted that in comparison to *CEBPA*-double mutants and *NPM1* mutants, children with AML who harbored *FLT3/ITD* or a *WT1* mutation and *FLT3/ITD* exhibited shorter telomeres. Finally, the presence of the high-risk molecular aberration *FLT3/ITD* was linked to shorter TL, but not to OS (140).

##### 4.4.1.2 Acute lymphoblastic leukemia

Several studies have reported shorter TL in blood samples of both adult and pediatric patients with newly diagnosed ALL compared to controls (141–143). Furthermore, it seems that TL is shorter to relapse patients vs newly diagnosed ALL patients vs healthy controls (143). TA is significantly elevated in ALL patients when compared to normal controls. The average TA level is reported higher in relapsed patients vs newly diagnosed vs control group (143).

TL is shorter in T-ALL vs B-ALL patients (133), and in adult vs pediatric ALL patients (141). Importantly, across all the AL subtypes, B-ALL cells showed the shortest telomeres and the highest TA (133). Male individuals have been reported to have higher TA levels than female individuals (144).

According to Wang et al, patients with high TA had a higher ratio of unfavorable cytogenetic abnormalities in ALL patients such as t(9;22), t(4;11), and t(11;14), whereas patients with low TA had a higher ratio of normal and other abnormalities (p < 0.05). In the same study, it was demonstrated that ALL subjects with high TA showed a significantly worse OS (31.65%) versus ALL subjects with low TA (58.82%, p < 0.01) (143).

###### 4.4.1.2.1 Pediatric acute lymphoblastic leukemia and telomeres

Borseen et al. reported that TL is significantly shorter in pediatric ALL at diagnosis compared to TL and end of therapy. Furthermore, TL was shorter in patients with *t(9;22)(q11;q34)[BCR/ABL1]* compared to high-hyperdiploid (51-61 chromosomes) or *t(12;21)* subjects and also compared to all B-cell precursor-ALL (BCP). Moreover, in comparison to BCP-ALL cases, hTERT methylation was more prevalent in T-ALL ones (18% compared to 72%). Moreover, BCP individuals with *t(12;21)* showed a significantly higher methylation rate (63%) compared to high-hyperdiploid BCP cases (7%). These findings are a strong indication that hTERT promoter methylation status may define certain ALL subgroups and have functional relevance. Finally, patients with BCP-ALL and short telomeres had a better prognosis (145).

#### 4.4.2 Chronic leukemias

##### 4.4.2.1 Chronic myeloid leukemia

The main characteristic of the myeloproliferative neoplasm named chronic myeloid leukemia (CML) is an uncontrolled malignant proliferation of myeloid cells in peripheral blood and bone marrow. The indolent chronic phase (CP), which lasts almost 3-5 years and has a notable increase in myeloid precursors and mature cells, is the first phase of CML’s natural triphasic course. CP proceeds to an accelerated phase (AP) if left untreated within a median time of 3-15 months, and then further progresses to a highly aggressive blast phase (BP). BP is characterized by a rapid expansion of primitive cells in BM that escape into the circulation. Philadelphia chromosome (Ph), which results from the reciprocal translocation t(9;22)(q34.1;q11.2) is a hallmark of CML (146).

TL in CML patients is shorter when compared to healthy individuals (146–149). In retrospective studies, it was demonstrated that accelerated telomere shortening was associated with the stage of disease, cytogenetic remission status, progression to accelerated phase, blast crisis, as well as clinical risk score at diagnosis (150).

Furthermore, average TL has been shown to be adversely associated with the progression of the disease, thus individuals in AP and BP present considerably shorter telomeres compared to patients in CP (147). Since 2000, it was shown that subjects in CP who eventually developed BP within 2 years had considerably shorter telomeres versus those who did not experience BP development for at least 2 years (PF 0.05) (148).

Increased expression of telomerase has been found to associate with an accumulation of additional cytogenetic abnormalities (151). Also, it has been suggested that patients with a more favorable prognosis may have longer telomeres (148).

Wenn et al. reported that CML patients with the longest telomeres showed a lower clinical risk profile compared to patients with shorter telomeres, thus implying that TL analysis may predict response to initial treatment with TKIs after 12 and 18 months according to ELN criteria (152).

In a recent study of 96 individuals with CP-CML, Estrada et al. found that patients with longer age-adjusted telomeres at diagnosis were more likely to achieve deep molecular response with imatinib when compared to individuals with shortened telomeres. Furthermore, major molecular responses were achieved remarkably earlier in subjects with long telomeres (p=0.012) (153).

##### 4.4.2.2 Chronic lymphoblastic leukemia

High telomerase expression and activity are found in CLL subjects with short telomeres, presumably to preserve the critical telomere length for cell survival (154).

The majority of CLL cases were found to keep a rather stable telomere length, nevertheless a subset of follow-up samples, especially those undergoing clonal evolution showed changes in TL (155).

Moreover, markers of worse prognosis like unmutated IGHV genes, increased genomic complexity, 11q deletion/mutated ATM gene, 17p deletion/mutated TP53 gene (155–157), as well as short time to first treatment (157) and short OS have all been linked to short telomeres (155, 157). According to Rambazzo et al, CLL subjects with 11q, 17p deletion, or 12 trisomy had considerably higher levels of telomerase and shorter telomeres, compared to those with no chromosomal abnormalities or only 13q deletion (P<0.001). In the same study, TL/telomerase level profiles were able to identify subgroups of patients with distinct clinical outcomes (P<0.0001) (158).

Of notice, Olbertova et al. reported substantially longer RTLs in individuals at Rai and Binet early disease stages at diagnosis (155). Longer telomeres in CLL patients were associated with improved outcomes compared to short telomeres (159), and significantly better prognosis in those with also mutated IGVH compared to CLL patients with short TL and unmutated IGVH (158).

### 4.5 Implications for treatment/discussion

The discovery of telomeres and telomerase has provided only a beginning in understanding the basic elements of the cell cycle and of several diseases with either shorter or longer telomeres that were previously considered idiopathic. Nowadays, studies have shown that multiple genetic and nongenetic factors might impact telomere length, thus it is not yet fully understood how these factors may interact. While genetic mutations involved in telomere length maintenance mechanisms are still being identified, it is more than obvious that additional studies are needed. One of the most important challenges is determining whether the combination of different factors-clinical, genetic, diseases specific- with telomere length may aid in identifying specific disease groups with different characteristics in clinical behavior, response to treatment, progression risk, and outcome.

Finally, while mechanisms of aging and cancer remain challenging in understanding, insights into telomeres and telomerase activity lead researchers to examine the possibility of potential drugs targeting those mechanisms (160). The assumption that inhibition of telomerase could serve as a promising therapeutic approach remains a challenge with several factors currently under investigation.

## Author contributions

KR: Conceptualization, Data curation, Formal analysis, Writing: review and editing. ES: Conceptualization, Writing: review and editing, Supervision, Funding acquisition, AK: Conceptualization, Writing: review and editing, Supervision, Funding acquisition. All authors contributed to the article and approved the submitted version.
